# The Involvement of Immune Semaphorins in the Pathogenesis of Inflammatory Bowel Diseases (IBDs)

**DOI:** 10.1371/journal.pone.0125860

**Published:** 2015-05-15

**Authors:** Zahava Vadasz, Tova Rainis, Afif Nakhleh, Tharwat Haj, Jacob Bejar, Katty Halasz, Elias Toubi

**Affiliations:** 1 Division of Allergy and Clinical Immunology, Bnai Zion Medical Center, Technion, Haifa, Israel; 2 Division of Gastroenterology, Bnai Zion Medical Center, Technion, Haifa, Israel; 3 Department of Pathology, Bnai Zion Medical Center, Technion, Haifa, Israel; Indiana University School of Medicine, UNITED STATES

## Abstract

**Background and Aims:**

Immune semaphorins are a large family of proteins involved in the pathogenesis of inflammatory diseases through the regulation of immune homeostasis and tissue inflammation. We aim to assess the possible involvement of semaphorin3A (sema3A) and 4A (sema4A) in peripheral immune responses and bowel tissue inflammation of patients suffering from Crohn’s disease (CD) and ulcerative colitis (UC).

**Patients and Methods:**

Twenty-seven CD patients and 10 UC patients were studied and compared to 10 patients followed for acute diverticulitis (disease control) and 12 healthy individuals. All were evaluated for sema3A expression on T regulatory cells (Tregs), serum levels of sema3A and sema4A, and tissue expression of sema3A and sema4A in bowel biopsies.

**Results:**

The percentage (%) of T regulatory cells (Tregs) expressing sema3A in patients with active CD (64.5% ±14.49%) and active UC (49.8% ±16.45%) was significantly lower when compared to that of healthy controls (88.7% ±3.6%, p< 0.001 and p< 0.0001, respectively). This expression was seen to be in negative correlation with CD activity. Serum levels of Sema4A were significantly lower in patients with CD and UC when compared to that of controls (5.69±1.48ng\ml for CD, 5.26±1.23 ng/ml for UC patients vs 9.74±2.73ng/ml for normal controls, P<0.001). Sema4A was highly expressed in lymphocytes of the lamina propria of CD and UC patients but absent in patients with diverticulitis or in normal individuals.

**Conclusions:**

Altered % of Tregs expressing sema3A in patients with inflammatory bowel diseases (IBD) is partially responsible for their failure in preventing CD4+ effector T cell induced inflammation in IBD in peripheral blood. The increased expression of sema4A in bowel biopsies from CD and UC patients is suggestive of its central role in regulating local tissue inflammation in the bowel.

## Introduction

Both Crohn’s disease (CD) and ulcerative colitis (UC) are inflammatory bowel diseases (IBDs), yet, their pathogenesis is totally different. Each has its own typical clinical, endoscopic and histopathological features. However, in both diseases, T cell over-activation is well documented, and T-cell regulatory functions are down-regulated and fail to suppress T effector cell responses. In CD, increased Th1 and Th17 cell activation is an important factor in inducing the inflammation in bowel lamina propria. Both types of T effector cells do react to bacterial bowel antigens when primary local immune responses fail to eliminate these antigens, and were therefore, found to be continuously exposed to adaptive immune responses. This situation leads to the intensive and continuous production of pro-inflammatory cytokines such as Th1 mediated ones, namely Interleukin (IL)-1, IL-6, TNF, and others [1–3].

As in most autoimmune and immune mediated diseases, the proper function of regulatory cells, namely T regulatory cells (Tregs), was also proven to be crucial in controlling CD. Treg cells regulate Th1 and Th2 differentiation and function by suppressing both cell responses through a direct cell-to-cell mechanism, the expression of membrane regulatory molecules of which CTLA-4 is the most important. The regulatory function of Treg cells is also mediated by expressing FoxP3, a "master regulator" and intracellular identification marker of Treg cells and the production of inhibitory cytokines, namely, IL-10 and TGF-beta [4, 5].

The involvement of Treg cells in CD is characterized by their altered FoxP3 expression, numbers, and function. CD4+CD25+FoxP3+ Tregs were found to be significantly lower in peripheral blood of patients with active CD, compared to normal individuals. In other studies, the altered function of Treg cells in IBD patients was more recognized, albeit in some cases their number was comparable. However, when these cells were assessed in bowel biopsies of these patients, both FoxP3 mRNA and Tregs were found to be highly expressed in the inflamed lamina propria, contrary to their expression in normal biopsies. FoxP3+Treg cells are part of the normal cellular compartment of the lamina propria and mesenteric lymph nodes (MLN). They were found to be significantly expanded in mucosal lymphoid tissues in CD, and to accumulate in T-cell-rich areas of MLN. The suppressor activity of FoxP3+ Treg cells is cell-to-cell dependent but also works by suppressing both Th1 and Th2 cytokines. However, in IBD patients, in attempt to maintain normal homeostasis and to hinder as much as possible CD4+ T effector cell induced inflammation, they remain unable to counterbalance the process of mucosal inflammation [6, 7]. A novel FoxP3 mutation (a missense variant in axon 6 of the x-linked FoxP3 gene) was recently reported in IBD patients. The mutation significantly reduced Treg suppressive abilities, but did not impair FoxP3 protein expression [8].

The issue of defining new regulatory molecules in various autoimmune or immune mediated diseases both membrane-bound and soluble has been the subject of many studies during the last two decades. In this regard, semaphorins (some of which play a role in the process of angiogenesis), were the favorite assessment candidates. Semaphorins were first described as playing a role in axonal guidance and neuronal development. They were soon understood to be frontline players in the field of immune responses. The role of semaphorin3A (sema3A) as being mainly a regulatory protein, was assessed by many studies. In an early one, it was elegantly shown that when soluble sema3A-Fc with or without sema3A antagonist was added to a culture of allogeneic dendritic cells (DC's) and T cells, it decreased their proliferation (measured by thymidine incorporation), but when these proteins were neutralized by specific antibodies, the proliferation of these T cells increased again [9]. In a later study, sema3A was found to be altered on CD4+ T cells of patients suffering from rheumatoid arthritis. Here, it was seen that sema3A was expressed only on CD4+NP-1+ T cells (Treg cells) and not on NP-1(negative) T cells. The co-culture of soluble sema3A with CD4+NP-1+ T cells increased their IL-10 production and their suppressive action on CD4+ T cell proliferation. In this same study, multiple injections of sema3A into the model mice of collagen-induced arthritis produced almost full remission and a decrease in the level of pro-inflammatory cytokines [10]. Sema4A is another member of this family thought to have a dual function. On one hand, it contributes to the activation of CD4+ T effector cells. When sema4A binds its receptor, it co-stimulates antigen-specific CD4+ T cell activation and leads to an increased IL-2 production. Sema4A has two known receptors, immune cell receptor Tim-2, the expression of which is highly restricted to activated T cells, preferentially to Th2 cells, and nonimmune cell receptor Plexin D1. Of note is that sema4A is expressed on dendritic and B cells but not T cells in a steady-state (normal) [11,12]. On the other hand, sema4A was also shown to be a potent regulatory molecule, reported to control Th2 cell-mediated inflammation. In this respect, allergen-treated sema4A-/- mice were shown to demonstrate an enhanced inflammatory response associated with increased interleukin 13 (IL-13), augmented airway hyper-reactivity, and lower Treg cell numbers in broncho-alveolar lavage of these mice. Lung resident cells including dendritic cells and eosinophils were isolated from the BAL of WT mice or from sema4A-\- mice, following the generation of in-vivo bone marrow chimera assay and AHR induction. It was concluded that chimera of sema4A-\- and WT improved the allergic reaction following induction of AHR, namely fewer eosinophils and Th2 cytokines. In a later study, sema4A was shown to control Th2-type inflammation by an in vivo systemic administration of sema4A-Fc to OVA sensitized mice, inhibiting by this IL-4-producing OVA-specific CD4 (+) T cells [13, 14].

The status and role of the above two semaphorins in IBDs has never been assessed. In this study our aim is to assess the expression of sema3A on Treg cells—known to be a regulatory factor in many autoimmune diseases—of CD and UC patients and to see if the expression changes in these cases. We also sought to evaluate whether serum levels of sema3A and sema4A in patients with IBDs change in the same manner in which they are altered in other immune mediated diseases. Finally, the involvement and status of these semaphorins in the histopathology of IBD was also evaluated.

### Patient population

The study participants were the following IBD patients: 15 patients with CD defined to be active, 12 patients defined as being in remission, and 10 patients with active UC. Ten patients suffering from acute diverticulitis served as disease controls. All patients were in routine follow-up at the Department of Gastroenterology in Bnai-Zion Medical Center. In addition, 12 healthy individuals served as normal controls. Blood was drawn from all participants and biopsies were performed at disease onset after receiving participants' signed informed consent and the approval of our local Helsinki committee. CD activity was assessed by using the CD activity index (CDAI) [15]. Remission was declared when the CDAI was less than 220 points and considered as flaring up when the CDAI was higher than 220 points. Table 1 summarizes the patient populations' clinical parameters.

**Table 1:** Clinical characteristics of Crohn’s disease patients.

*CRP (mgr/L)*	*CDAI*	*Sema3A expression on CD4* ^+^ *CD25* ^+^ *(%)*	*Gender*	*Age (Yr)*	*Patient Num*.
				***Active Crohn’s***	
80.7	252	66.3%	Female	40	1
14	222	65.9%	Male	28	2
222.3	358	74.5%	Male	26	3
249.3	328	73.6%	Female	46	4
33.4	215	64.1%	Female	55	5
123.4	339	75%	Female	21	6
17	220	59%	Female	23	7
13.4	244	78.2%	Male	22	8
208.6	389	69%	Male	24	9
73.8	302	68%	Male	70	10
102.3	292	74%	Female	38	11
219.7	324	52.2%	Male	36	12
61	289	63.8%	Female	69	13
81.6	301	57.1%	Male	27	14
19	267	74%	Female	40	15
				***Silent Crohn’s***	
1	66	86.7%	Female	62	16
3.6	41	84.6%	Female	44	17
5.8	32	90.4%	Male	52	18
1.2	35	87%	Male	30	19
16.9	80	79%	Male	28	20
14.7	110	82%	Male	29	21
4.5	56	78.2%	Female	59	22
2.9	44	77.1%	Female	47	23
0.7	59	59.1%	Female	40	24
3.4	41	64.2%	Female	62	25
8	69	65.1%	Female	55	26
0.8	38	77.7%	Female	33	27

## Material and Methods

### 1. Serum levels of sema3A and 4A

Serum samples were drawn from all studied individuals during the study and were stored at -20° until analyzed together by ELISA. We used commercial ELISA kits for both semaphorins (MBS-MyBiosource, San Diego, California, USA: for sema3A we used the MBS160764 kit, for sema4A we used the MBS940246 kit). Assessment was performed according to the manufacturer’s instructions.

### 2. Treg cells expressing sema3A

The percentage (%) of Tregs (CD4+ CD25+high T cells) expressing sema3A in all studied groups (e.g., CD patients at remission and at relapse, UC patients at disease onset, patients suffering from acute diverticulitis and normal healthy controls) was performed on purified mononuclear cells, gating on CD4+CD25high cells and staining them with monoclonal antibodies: human anti-CD4 FITC /PE and CD25 PC5 (Immunotech, Beckman-Coulter, Marseille, France), and human anti-sema3A AlexaFluor 488 (R&D, Minneapolis, MN, USA), and evaluated using flow cytometry software (FC500 and CXP software, Beckman Coulter, Brea, CA, USA). The results are shown as % of Treg cells expressing sema3A, taking into consideration that the absolute number of Treg cells in all groups was found to be comparable. We defined the threshold of Treg cells expressing sema3A as the one above 2.5 Mean-Fluorescent-Intensity (MFI) (Beckman- Coulter FACS, FC-500 Software). Standard deviation (STDEV) was used to quantify the amount of variation of a set of data values (e.g. percentage of Treg cells expressing sema 3A among the patients in each indicated group of disease or normal control).

### 3. Detection of sema3A and sema4A expression in biopsies of IBD patients

#### 3.1 Reagents

Anti-human sema3A and 4A antibodies were obtained from “Abcam” (Cambridge, UK). The “histostain+” immunohistochemistry kit was purchased from Invitrogen (Camarillo, CA, USA).

#### 3.2 Immunohistochemistry

Formalin-fixed retrospectively paraffin-embedded colon tissue sections were taken from the archive of the Department of Pathology, Bnai-Zion Medical Center. These biopsies were taken from 20 CD and 10 UC patients at diagnosis onset. In addition, 10 biopsies were taken from acute diverticulitis patients, as disease controls, along with 10 normal colon biopsies. The biopsies were serially sliced into 5μm slices by a microtome. The slides were deparaffinized by xylene and then rehydrated by absolute ethanol, 96% ethanol, 70% ethanol and two quick distilled water washes. The antigen retrieval was done by cooker pressure in an EDTA solution (2mM, and pH-8.0). H_2_0_2_ 3% in methanol was used to block endogenous hydrogen peroxidase. In order to block non-specific endogenous biotin we used a commercial biotin blocker, according to manufacturer’s instructions. Primary antibodies were incubated with the slides as follows: Anti-human sema3A, 4A (1:100), anti-human CD19, CD3 (1:50). Secondary antibody and staining were done by using the commercial “Histostain+” kit, according to manufacturer’s instructions. Slides that were used for specificity controls underwent the same procedure without primary antibodies. No staining was observed in these controls (data not shown). The immuno-histochemical staining was evaluated by two independent pathologists and was scored for localization and staining intensity. Staining intensity scores ranged from negative (scored as “0”) to the maximal positive value (scored as “3”). All slides from all studied individuals were given a staining score and the final score was the average intensity of all scores.

### 4. Clinical Correlations

The % of Tregs in CD patients was analyzed in correlation with their disease activity determined by the CDAI score. The level of sema4A in serum was also analyzed in respect to its correlation with the CDAI.

### 5. Statistical analyses

Comparison of % of Treg cells expressing sema3A and serum levels of sema3A and sema4A of IBD patients, acute diverticulitis patients and healthy controls was performed using the unpaired student t-test. The correlation coefficient (r) of clinical correlation between the CDAI score and sema3A expression was determined using the Pearson correlation test. A two tailed *P*-value of 0.05 or less was considered to be statistically significant.

## Results

### 1. The % of Tregs expressing sema3A

**Fig 1:**
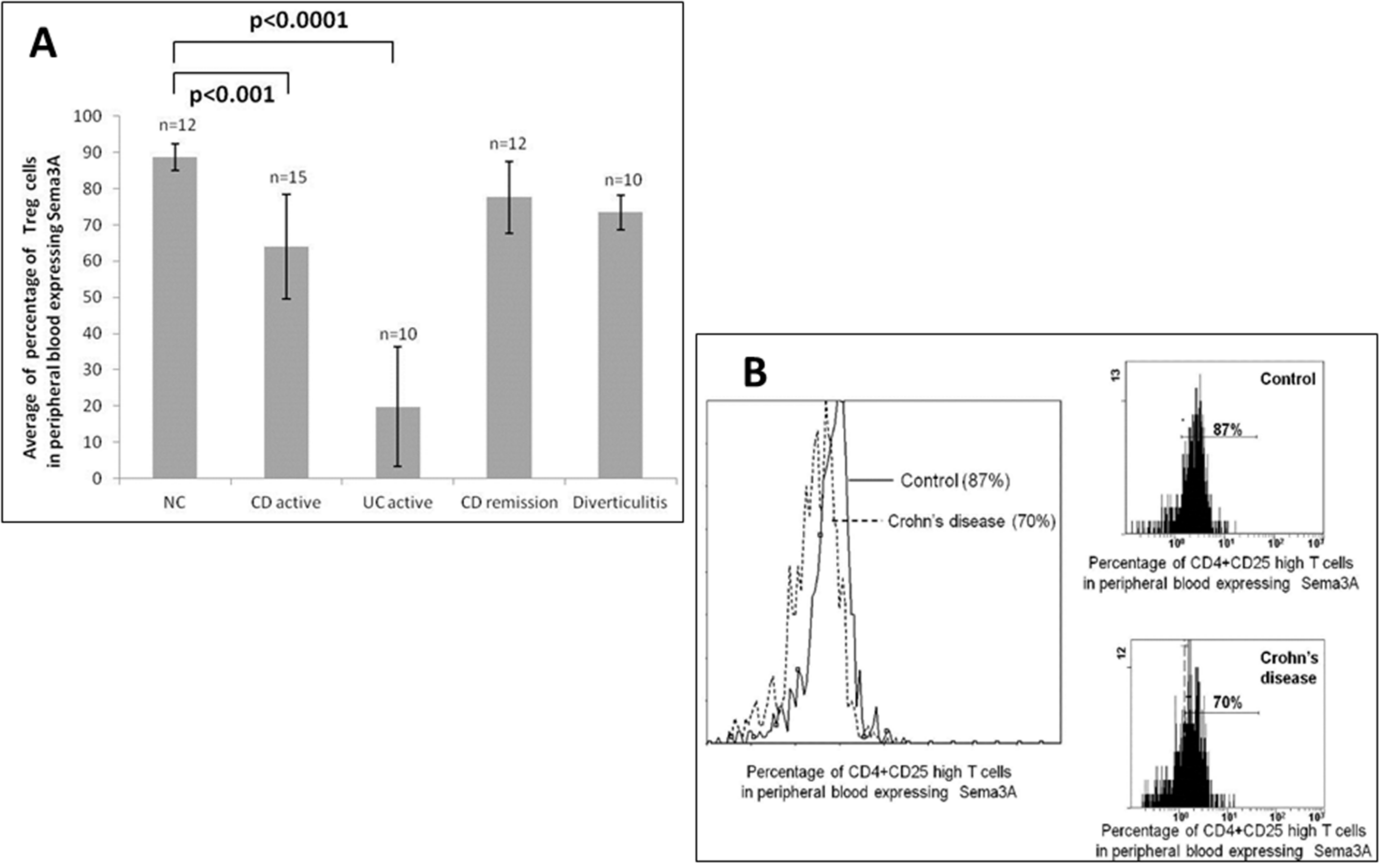
A: The percentage of Treg cells expressing sema3A. The percentage of Treg cells expressing sema3A in peripheral blood in patients suffering from Crohn’s disease [both active (n = 15) or in remission (n = 12)], ulcerative colitis (active, n = 10), and from patients suffering from acute diverticulitis (n = 10) compared to that from healthy controls (n = 12). Note the significantly altered percentage of Treg cells expressing sema3A in all IBD patients when compared to that of normal individuals. B: A representative figure of Treg cell expressing sema3A. A representative FACS analysis of Treg cell expressing sema3A in a CD patient compared to that of normal individual.

The percentage of Treg cells (CD4^+^CD25^high^ cells) in peripheral blood expressing sema3A was found to be significantly lower in patients with active CD (64.5%±14.49%) compared to 88.7%±3.6% in healthy individuals (p < 0.001). This was also found to be significantly lower in CD patients in remission (77.6% ±9.93%, p<0.001) and in patients with active UC and diverticulitis (49.8% ±16.45%, p<0.0001 and 73.9% ±4.79%, respectively) (see Fig 1A).

### 2. Correlation between the percentage of Treg cells expressing sema3A and CDAI score

**Fig 2:**
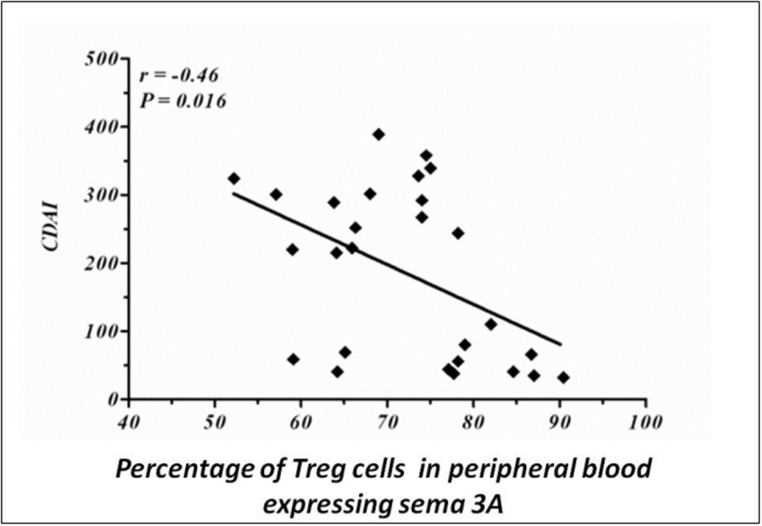
Clinical correlation. The percentage of Treg cells expressing sema3A in peripheral blood of CD patients is found to be in negative correlation with the CDAI score (r = -0.46, p = 0.016).

We further analyzed the clinical correlation between the percentage of Treg cells expressing sema3A taken from peripheral blood of CD patients and the CDAI score of these patients. As is shown in Fig 2, there is a statistically significant negative correlation between these two variables (r = -0.46, P = 0.016). However, when a clinical correlation between the CDAI index and sema3A was calculated for each patient's group, we could find only a statistical trend.

### 3. Serum levels of sema3A and sema4A

**Fig 3:**
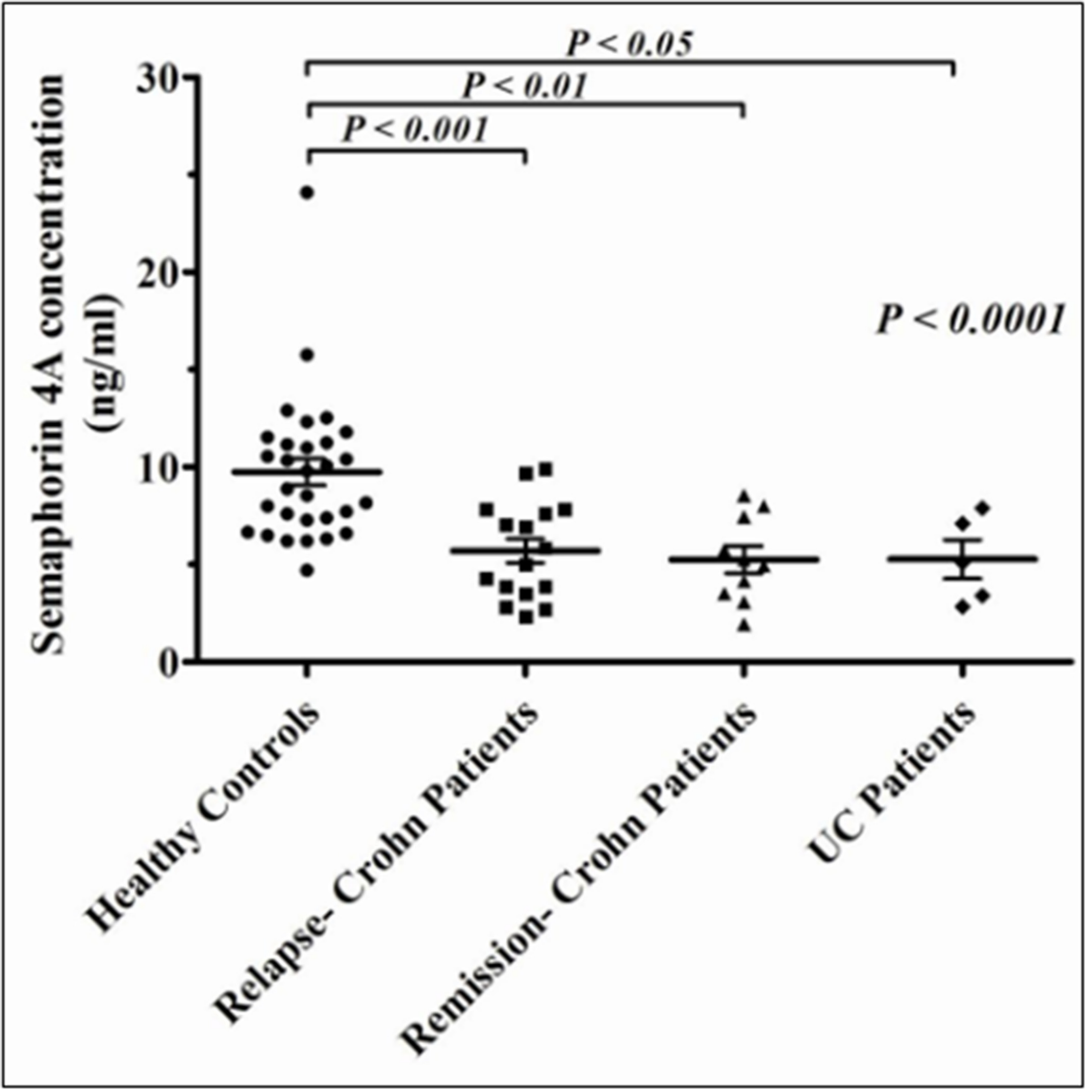
Serum levels of sema4A. Serum levels of sema4A are significantly lower in CD (in active disease, n = 15) and UC patients (n = 10), when compared to the level of sema4A in normal individuals (n = 30).

Serum levels of sema3A were found to be mildly lower in CD and UC patients but with no significant statistical difference when compared to that of healthy individuals (data not shown). However, as is demonstrated in Fig 3, the level of sema4A serum was significantly lower in both IBD groups (similarly low in both CD and UC patients) when compared to that of healthy individuals (5.69±1.48ng/ml for CD, 5.26±1.23 ng/ml for UC patients vs 9.74±2.73ng/ml for normal controls, P<0.001). We could not see any correlation between the level of sema4A serum and CD activity.

### 4. Immunohistochemistry

**Fig 4:**
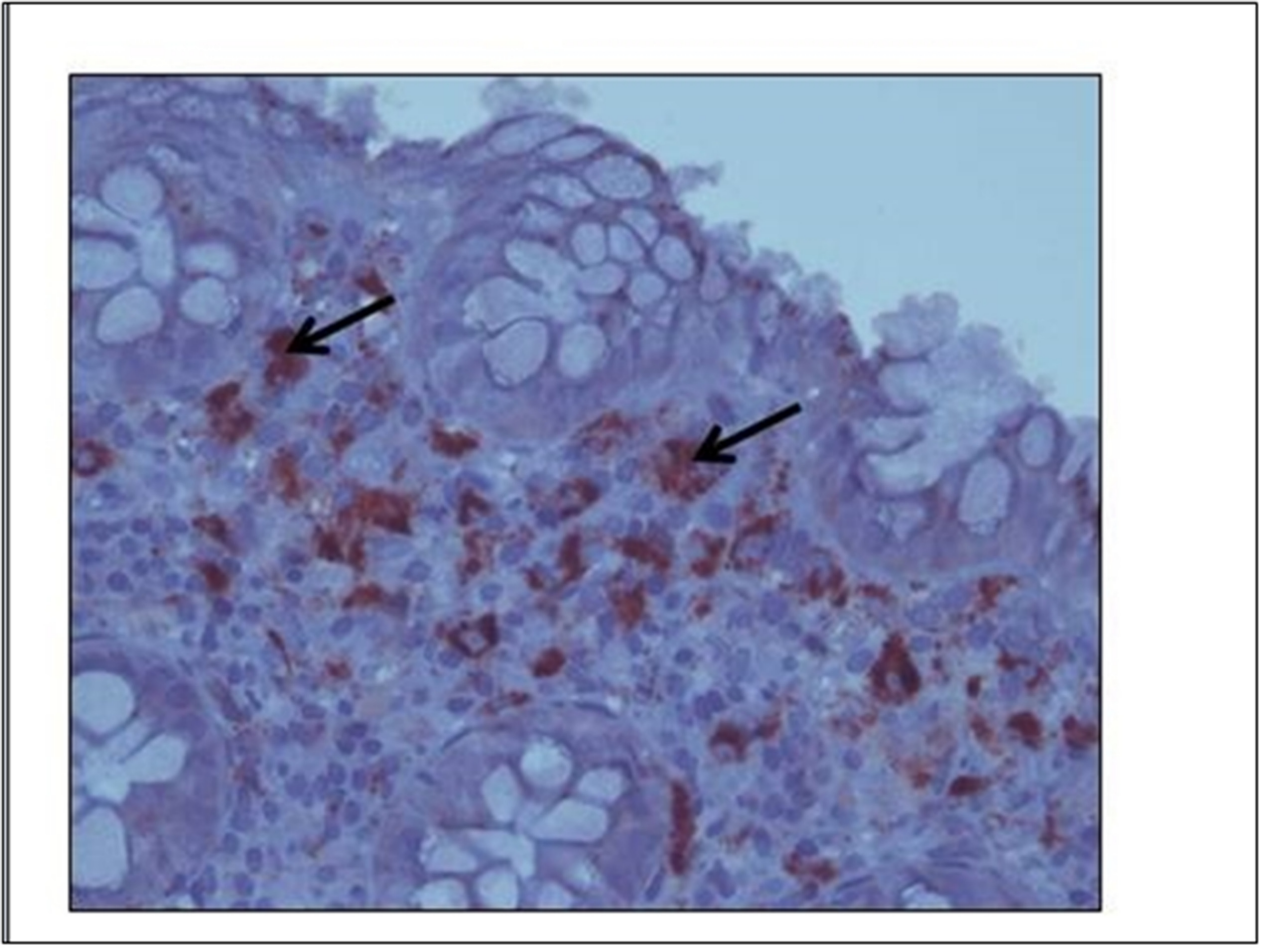
Sema3A staining in IBD. A representative biopsy from an active CD patient in which sema3A is intensely stained (+3) in the macrophages of the lamina propria (similarly in all studied groups). Black arrows denote positively stained macrophages.

**Fig 5:**
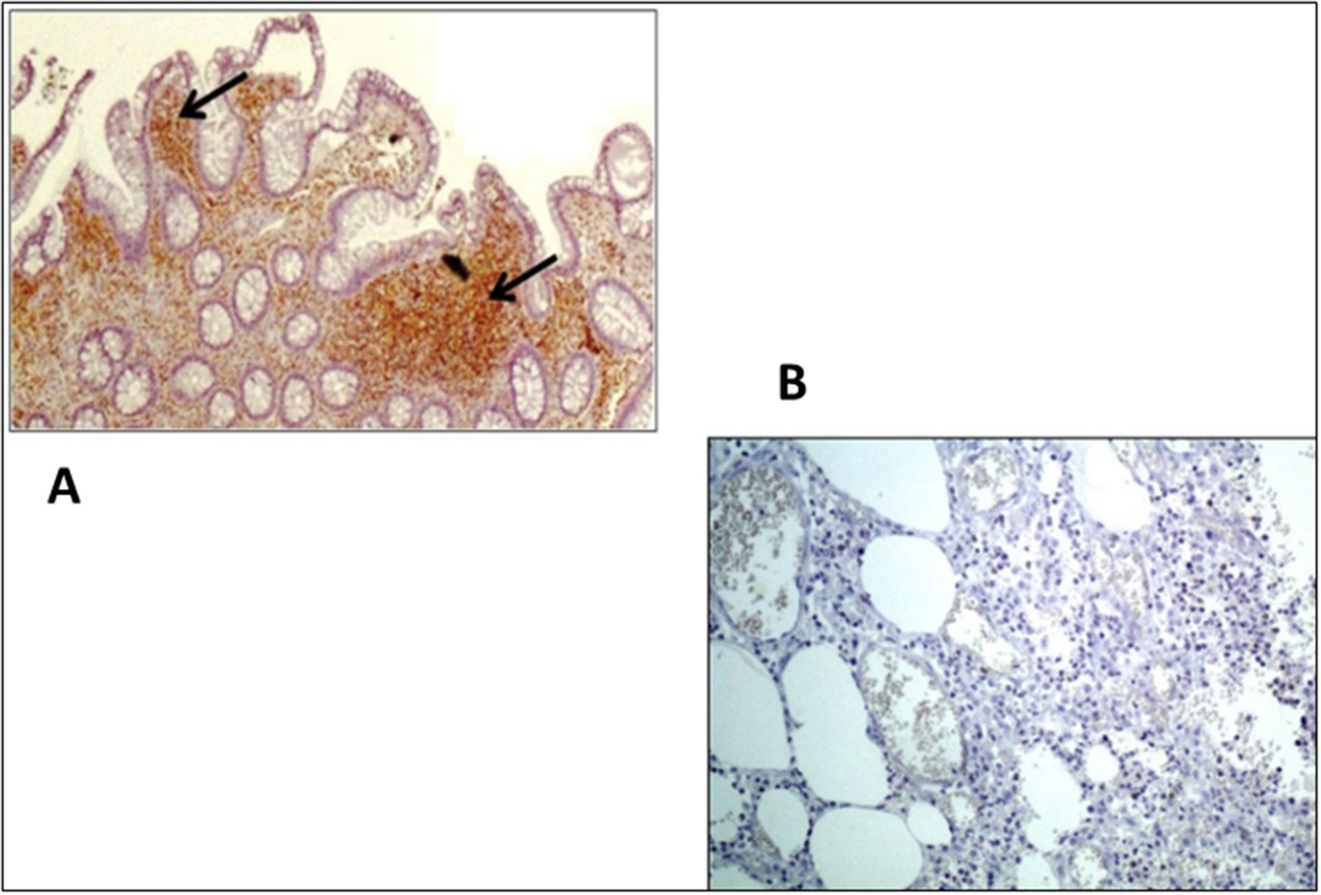
Immunohistochemistry staining of sema4A. Sema4A is clearly detected in lymphocytes of the lamina propria in a biopsy taken from a CD patient (**A**), but was not detected in a biopsy taken from a diverticulitis patient (**B**) or from a normal individual. Black arrows denote positively stained lymphocytes.

We analyzed the expression pattern of the immune semaphorins—namely, sema3A and sema4A in colon biopsies taken from all IBD patients at onset of disease, from patients suffering from diverticulitis and from normal controls. As is shown in Fig 4, the pattern of different semaphorin expressions was as follows: Fig 4, **Sema3A**—the expression pattern was found to be similar in all four studied groups. It was found to be highly expressed (+3) in the macrophages and with medium intensity (+2) in the lymphocytes of the lamina propria. Neuroendocrine cells in the cripts were also stained positively. Fig 5, **Sema4A**—medium intensity expression (+2) was detected in lymphocytes of the lamina propria in biopsies taken from CD and UC patients (A), but was not detected in biopsies taken from normal controls or from diverticulitis biopsies (B).

## Discussion

The focus on understanding the wide spectrum of pro-inflammatory mechanisms in the pathogenesis of autoimmune/immune mediated diseases was the main issue in most studies for many decades. These mechanisms included cellular and humoral immune responses followed by the production of pro-inflammatory cytokines such as TNF, IFNs, IL-17 and others. They also included the overexpression of co-stimulatory molecules such as CD 86, and CD40L on effector T cells and the increased production of B cell activating factor (BAFF) [16–18].

During the last decade, attention has shifted to the appreciation of regulatory immune responses, the normal function of which was proven to suppress autoimmunity and maintain self-tolerance. Among the early regulatory molecules that researchers found, are T regulatory cells, and the production of inhibitory cytokines such as IL-10 and TGF-beta and their function in maintaining self-tolerance [19]. However, as the interest in these mechanisms increased, other regulatory responses were seen, such as the role of B regulatory cells and the participation of regulatory semaphorins in maintaining immune mediated homeostasis. In a previous study of ours, we assessed the possible involvement of sema3A, sema4A and sema4D in contributing to or regulating glomerular inflammation in lupus nephritis. In this earlier study, sema3A expression was not seen in the glomeruli but was strongly expressed in the tubuli, suggesting that it is a marker for tubular damage in SLE. Sema4A, on the other hand, was found to be expressed both in glomeruli and tubuli, and was noticed to be inversely correlated with the extent of renal damage. We, therefore, suggested that sema4A is locally produced, aiming to suppress inflammation. In this respect we also raised the possibility that the local production of immune sema4A and sema3A is gradually altered in advanced glomerular damage, and is, therefore, minimally expressed in diffuse glomerulonephritis [20]. With this in mind, we further reported that serum levels of sema3A were significantly lower in patients with systemic lupus erythematosus when compared to that in normal individuals. In addition, co-culture of activated B cells of SLE patients with sema3A reduced the expression of TLR-9 in these B cells, thus suggesting that sema3A is a modulator in reducing autoimmunity [21]. This study stirred our interest in assessing the role of immune semaphorins in other immune mediated disorders.

The present study is the first one to show that sema3A is involved in immune responses of both CD and UC, by demonstrating that the % of Tregs expressing sema3A in peripheral blood is significantly decreased in these diseases. This was noticed to be in negative correlation with CD activity. This status of Tregs, is suggested to be responsible for their failure to inhibit Th1 over-activity, thus contributing to the process of inflammation. However, the fact that soluble sema3A in the sera of these patients was comparable to that of normal individuals, and was similarly expressed in the lamina propria of all studied groups, raises the possibility that sema3A plays a crucial role in regulating some aspects of peripheral blood phases of inflammation but of less importance in later phases (tissue inflammation).

Our main important finding in this study is the increased expression of sema4A in infiltrating lymphocytes of the lamina propria in both active CD and UC. It is theorized that this increased expression becomes a histopathological marker for these diseases, being absent in the lamina propria of patients with acute deverticulitis and of healthy individuals, which may be established in future studies.

In many studies, sema4A is reported to be important for Th1 differentiation after T cell receptor stimulation. However, in a very recent study, the immune-cell expressed ligand sema4A and Treg-cell expressed receptor neuropilin-1 (Nrp1) were elegantly shown to interact both in vitro-to potentiate Treg cell function and their survival- and also in vivo—at inflammatory sites such as in our case, at the inflamed bowel [22].

With this in mind we may assume that our finding of lower serum levels of sema4A in IBD patients (the precise explanation of which is not fully understood) is partially contributing to the failure of Treg cells in inhibiting CD4+ T cell pro-inflammatory responses. It is possible that increased sema4A expression in biopsies taken from active IBD patients is dispensable for suppression of immune-mediated, CD4+ effector T cell over-activity and maintenance of immune homeostasis. In line with this hypothesis, we propose that this enables the enhancement of Treg cell activity in the inflamed sites of the bowel, in an attempt to decrease established inflammatory colitis. Sema4A ligation of Nrp1 increases nuclear localization of transcription factor FoxP3. This is in agreement with previous findings where Treg cells and Foxp3 were also found to be overexpressed in the bowels of CD patients, suggesting this is a regulatory compensation in the inflammation process in IBD [6, 7]. One can speculate that sema4A co-presence with Treg cells in the inflamed bowel could possibly be a regulatory block, decreasing by this CD4+ T cell activation, and therefore, becomes a frontier regulator in active IBD, while at the same time, also a marker for disease activity.

The answer to the questions of the origin of sema4A being reduced in the sera of IBD patients and whether sema4A is produced locally in inflamed sites is not yet clear. Finally, the possible benefit of targeting these semaphorins, implying a new therapeutic direction in treating IBDs should be evaluated.
